# Phase I study assessing the mass balance, pharmacokinetics, and excretion of [^14^C]-pevonedistat, a NEDD8-activating enzyme inhibitor in patients with advanced solid tumors

**DOI:** 10.1007/s10637-020-01017-x

**Published:** 2020-10-22

**Authors:** Xiaofei Zhou, Farhad Sedarati, Douglas V. Faller, Dan Zhao, Hélène M. Faessel, Swapan Chowdhury, Jayaprakasam Bolleddula, Yuexian Li, Karthik Venkatakrishnan, Zsuzsanna Papai

**Affiliations:** 1 Millennium Pharmaceuticals, Inc., a wholly owned subsidiary of Takeda Pharmaceutical Company Limited, 40 Landsdowne Street, Cambridge, MA 02139 USA; 2 Medical Center Hungarian Defense Forces, Budapest, Hungary

**Keywords:** pevonedistat, advanced solid tumors, phase I, mass balance, pharmacokinetics, elimination

## Abstract

Pevonedistat (TAK-924/MLN4924) is an investigational small-molecule inhibitor of the NEDD8-activating enzyme that has demonstrated preclinical and clinical activity across solid tumors and hematological malignancies. Here we report the results of a phase I trial characterizing the mass balance, pharmacokinetics, and clearance pathways of [^14^C]-pevonedistat in patients with advanced solid tumors (NCT03057366). In part A (*n* = 8), patients received a single 1-h intravenous infusion of [^14^C]-pevonedistat 25 mg/m^2^. In part B (*n* = 7), patients received pevonedistat 25 or 20 mg/m^2^ on days 1, 3, and 5 in combination with, respectively, docetaxel 75 mg/m^2^ or carboplatin AUC5 plus paclitaxel 175 mg/m^2^ on day 1 every 3 weeks. Following the single dose of [^14^C]-pevonedistat 25 mg/m^2^ in part A, there was a parallel log-linear decline in plasma and whole blood pevonedistat concentration, with systemic exposure of unchanged pevonedistat representing 41% of drug-related material (i.e., unchanged pevonedistat and its metabolites). The mean terminal half-life of pevonedistat and drug-related material in plasma was 8.4 and 15.6 h, respectively. Pevonedistat distributed preferentially in whole blood with a mean whole-blood-to-plasma ratio for pevonedistat AUC_∞_ of 40.8. By 1 week post dose, the mean recovery of administered radioactivity was 94% (41% in urine and 53% in feces). The pevonedistat safety profile during both study parts was consistent with previous clinical experience, with no new safety signals observed. In part B, pevonedistat in combination with docetaxel or carboplatin plus paclitaxel was generally well tolerated. ClinicalTrials.gov identifier: NCT03057366.

**Electronic supplementary material:**

The online version of this article (10.1007/s10637-020-01017-x) contains supplementary material, which is available to authorized users.

## Introduction

Pevonedistat (TAK-924/MLN4924) is an investigational small-molecule inhibitor of the neural precursor cell-expressed, developmentally down-regulated 8 (NEDD8)-activating enzyme (NAE) [1, 2]. NAE conjugates NEDD8 to cullin-RING ligases (CRLs), which control ubiquitination and proteasomal degradation of substrates involved in cell cycle progression (p21, p27, and cyclin D/E), DNA replication (CDT1), oxidative response (NFR2), and response to hypoxia (HIF1a) [3, 4]. This conjugation process, called neddylation, activates CRLs to ubiquitinate and degrade their substrates [5]. Pevonedistat forms a covalent adduct with NEDD8 that binds tightly to NAE, preventing neddylation; this results in CRL substrate accumulation, which leads to apoptotic cell death [6]. Accumulating evidence also suggests that neddylation has a pivotal role in regulating immune cell function and tumor angiogenesis in the tumor microenvironment [7]. Moreover, pevonedistat also blocks prosurvival signaling by NF-κB [1, 8]. Dysfunction of the NEDD8 cascade is linked to the pathogenesis of several diseases, including cancer, making it a compelling target for drug development [7, 9].

Pevonedistat has demonstrated potent antitumor activity in preclinical studies, demonstrating growth inhibition across a variety of tumor cell lines including solid tumors (colon, lung) and hematological malignancies (myeloma, lymphoma) [6, 8, 10]. These data indicate that pevonedistat may have broad activity across numerous tumor types. Pevonedistat has also shown activity in tumor xenograft models in mice. In a preclinical analysis of mice bearing human xenograft tumors of activated B-cell (ABC) and germinal center B-cell (GCB) diffuse large B-cell lymphoma (DLBCL), in vivo administration of pevonedistat blocked NAE pathway biomarkers and resulted in complete tumor growth inhibition [8]. In human colon and lung tumor xenograft models in immunocompromised mice, a single dose of pevonedistat resulted in inhibition of NEDD8 conjugation and increased NAE inhibition, while repeated dosing effected tumor growth inhibition in these models [6]. In mice bearing acute myeloid leukemia (AML) xenograft models, pevonedistat resulted in durable disease regression and reduction of neddylated cullins [10].

Numerous phase I/Ib studies have examined the activity of pevonedistat alone or in combination with other agents in multiple tumor types, including advanced solid tumors, melanoma, AML, myelodysplastic syndromes, multiple myeloma, and lymphoma [2, 11–16]. In these studies, pevonedistat was shown to be clinically active and generally well tolerated in patients with advanced solid tumors or hematological malignancies [11, 13–16]. In the setting of advanced solid tumors, pevonedistat was assessed in combination with standard-of-care chemotherapy in patients whose disease had progressed despite standard therapy or for whom conventional therapy was ineffective [12]. The maximum tolerated dose (MTD) of pevonedistat was determined to be 25 mg/m^2^ in combination with docetaxel and 20 mg/m^2^ in combination with carboplatin plus paclitaxel. Pevonedistat was well tolerated in both combinations, with the most common treatment-emergent adverse events (TEAEs) including fatigue, nausea, constipation, diarrhea, vomiting, and anemia. Sustained clinical responses were observed in patients receiving pevonedistat in combination with carboplatin plus paclitaxel (overall response rate [ORR]: 35%), and the ORR in combination with docetaxel was 16%.

Previous studies in patients with solid tumors or hematological malignancies have evaluated the single- and multiple-dose pharmacokinetics (PK) of intravenous (IV) pevonedistat administered across a range of doses and dosing schedules [2, 11, 13, 14]. Plasma concentrations of pevonedistat were shown to decline in a bi-exponential manner at the end of a 1-h IV infusion, with little or no notable accumulation with once-daily dosing for 5 consecutive days or with intermittent dosing, which is consistent with the mean terminal elimination half-life of approximately 5–8 h estimated across doses and schedules. Pevonedistat PK was linear and dose-proportional over the dose range studied (25–278 mg/m^2^). Pevonedistat population PK was assessed using data from six studies of pevonedistat alone or in combination with current standard-of-care agents [17]. The pevonedistat PK profile was shown to be comparable in patients with solid tumors and hematological malignancies, and a clinically meaningful effect of body size on PK variability was identified, supporting the use of body surface area (BSA)-based dosing.

In vitro metabolism studies have suggested a major role for CYP3A in pevonedistat elimination pathways; however, strong and moderate CYP3A inhibitors did not result in clinically meaningful increases in pevonedistat exposure [23]. Here we discuss the results of a phase I study designed to characterize the mass balance, PK, and routes of elimination of [^14^C]-pevonedistat in patients with advanced solid tumors. The study comprised two parts: part A assessed mass balance, PK, metabolite profiles, safety, and tolerability in patients who received a single IV infusion of [^14^C]-pevonedistat, while in part B, safety, tolerability, and disease response were assessed in patients who received treatment with pevonedistat in combination with either docetaxel or carboplatin plus paclitaxel.

## Methods

### Study design

This was a two-part (A and B), open-label, multicenter, mass balance, distribution, metabolism, excretion (DME) study in adult patients with advanced solid tumors. In part A, patients received a single IV infusion of [^14^C]-pevonedistat 25 mg/m^2^ (containing approximately 60–85 µCi, or 2.22–3.145 MBq, of total radioactivity) at a single center (Duna Medical Center, Budapest, Hungary). Patients were required to stay at the part A study site for approximately 8 days or until the discharge criteria were met, with a maximum estimated confinement period of 9–14 days. Upon completion of part A, patients had the option to participate in part B at Medical Center Hungarian Defense Forces, Budapest, Hungary. In part B, patients received treatment in 3-week cycles with either: pevonedistat 20 mg/m^2^ on days 1, 3, and 5 plus carboplatin AUC5 and paclitaxel 175 mg/m^2^ on day 1; or pevonedistat 25 mg/m^2^ on days 1, 3, and 5 plus docetaxel 75 mg/m^2^ on day 1. Treatment was administered for 12 cycles or until discontinuation criteria were met.

### Study objectives

The primary objectives of the study were to assess the mass balance (cumulative recovery of total radioactivity in urine and feces) and to characterize the PK of pevonedistat (whole blood, plasma, and urine) and total radioactivity (drug-related material; plasma and whole blood) following a single infusion of [^14^C]-pevonedistat 25 mg/m^2^ (part A).

The secondary objectives were: to profile and identify circulating and excretory metabolites in plasma, whole blood, urine, and feces; to evaluate the safety and tolerability of pevonedistat in patients with advanced solid tumors after a single dose (part A) and in combination with docetaxel or carboplatin plus paclitaxel (part B); and to evaluate disease response with the combination of pevonedistat and docetaxel or carboplatin plus paclitaxel (part B). Pevonedistat metabolite profiling and identification are not described herein.

### Patients

Patients aged ≥ 18 years were required to have histologically or cytologically confirmed metastatic or locally advanced and incurable solid tumors that had progressed despite prior standard therapy or for which conventional therapy was not considered effective. An Eastern Cooperative Oncology Group (ECOG) performance status of 0 or 1 and expected survival of ≥ 3 months were also required. The tumor must have been radiographically or clinically evaluable and/or measurable. Patients were not permitted to have received treatment with any systemic antineoplastic therapy or any investigational products within 21 days before the first dose of study treatment, or antibiotic therapy, radiotherapy, or major surgery within 14 days. Any patient who continued to part B was to be re-evaluated for entry criteria before treatment in part B could begin. Full inclusion and exclusion criteria can be found in the Supplementary Methods.

### Assessments

Blood samples were collected for total radioactivity and PK assessments in whole blood and plasma at prespecified time points (pre dose [within 1 h before the start of the pevonedistat infusion], end of infusion, then 0.5, 1, 2, 3, 4, 8, 12, 24, 48, 72, 96, 120, 144 and 168 hours post dose). Complete urinary and fecal output were collected for analysis of PK (urine) and total radioactivity (urine and feces) throughout the confinement period until discharge criteria were met. Bioanalytical assay methods are shown in the Supplementary Methods. Safety was assessed by incidence and severity of TEAEs, vital signs, physical examinations, electrocardiograms, and clinical laboratory tests, and toxicities were graded according to the National Cancer Institute Common Terminology Criteria for Adverse Events (NCI CTCAE), version 4.03. In part B, disease assessments were conducted using radiological evaluations (computed tomography [CT] scan or magnetic resonance imaging [MRI], as clinically indicated).

### Pharmacokinetic analysis

Non-compartmental analysis using Phoenix™ WinNonlin® version 8.0 (Pharsight Corp., Mountain View, California, USA) was used for estimation of PK parameters for pevonedistat in whole blood, plasma, and urine and for total radioactivity in whole blood, plasma, urine, and feces. The following single-dose PK parameters were derived: maximum plasma concentration (C_max_), area under the concentration–time curve from time 0 to infinity (AUC_∞_), AUC from time 0 to the last quantifiable concentration (AUC_0 − last_), terminal disposition phase half-life (t_1/2_), renal clearance (CL_R_), clearance (CL), and volume of distribution at steady state (V_ss_).

### Statistical analysis

PK was assessed in all patients who received the single 1-h [^14^C]-pevonedistat dose in part A, did not receive any excluded medications, and had sufficient concentration–time data to permit reliable estimation of PK and mass balance parameters (PK-evaluable population). PK data were summarized using descriptive statistics, and the PK and mass balance parameters described in Supplementary Table S1 were calculated by non-compartmental analysis for each individual patient. Safety was assessed in all patients who received at least one dose of pevonedistat, and TEAEs were coded according to the Medical Dictionary for Regulatory Activities (MedDRA), version 20.0. Analysis of efficacy in part B was descriptive; disease response was assessed in all patients who received at least one dose of pevonedistat, had measurable disease (per entry criteria for part B), and had at least one post-baseline disease assessment. Response assessment was done by the investigator using Response Evaluation Criteria in Solid Tumors (RECIST) version 1.1. SAS version 9.4 was used for all statistical analyses.

## Results

### Patients

Eight patients were enrolled in part A of the study (Table 1). All patients received at least one dose of pevonedistat and were included in the safety population. All 8 patients completed part A, and 7 patients were included in the PK-evaluable population; 1 patient was excluded due to use of a prohibited medication (carbamazepine) on day 2. All 8 patients consented to participate in part B of the study; however, 1 patient was excluded due to not meeting the entry criteria for part B. In part B, all 7 patients received at least one dose of study drug and were included in the safety population (pevonedistat/carboplatin/paclitaxel, *n* = 5; pevonedistat/docetaxel, *n* = 2).

**Table 1:** Demographics and baseline disease characteristics (safety population)

		Patients (*N* = 8)
Age, years	Mean (SD) Median (range)	61.8 (13.22) 64.0 (45–76)
Gender, *n* (%)	Male Female	3 (37.5) 5 (62.5)
Race, *n* (%)	White	8 (100)
Weight, kg^a^	Mean (SD) Median (range)	82.38 (15.777) 82.00 (65.0–112.0)
BMI, kg/m^2b^	Mean (SD) Median (range) Minimum, maximum	29.58 (4.674) 29.20 (22.9–39.6) 22.9, 39.6
BSA, m^2a^	Mean (SD) Median (range)	1.943 (0.2195) 1.960 (1.65–2.30)
Serum bilirubin, µmol/L^c^	Mean (SD) Median (range)	11.3 (4.23) 10.0 (8–21)
Creatinine, µmol/L^c^	Mean (SD) Median (range)	89.5 (16.40) 92 (59–112)
Alanine aminotransferase, U/L^c^	Mean (SD) Median (range)	21.6 (7.67) 23.5 (11–31)

*BMI*, body mass index; *BSA*, body surface area; *SD*, standard deviation

^a^Baseline measurements for weight and BSA were taken at Day −1

^b^Baseline measurements for BMI were taken at screening

^c^Baseline measurements for serum bilirubin, creatinine, and alanine aminotransferase presented in this table were taken at Day −1

### Drug exposure

All patients in part A received the planned dose of [^14^C]-pevonedistat 25 mg/m^2^ without modification or interruption. The actual administered dose ranged from 40 mg to 50 mg based on patient BSA. In part B, patients received a median of 5.0 treatment cycles of pevonedistat/carboplatin/paclitaxel and 3.5 cycles of pevonedistat/docetaxel. All patients received at least 2 cycles of treatment, and 2 patients in the pevonedistat/carboplatin/paclitaxel arm received 11 cycles.

### Pharmacokinetics

**Fig. 1:**
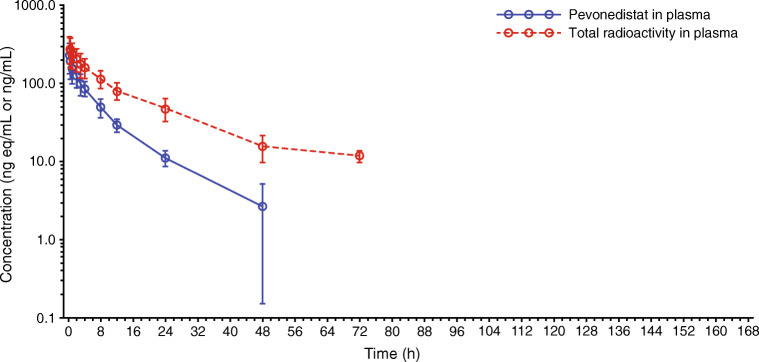
Mean concentration–time profiles of pevonedistat and total radioactivity (drug-related material) in plasma following a single infusion of [^14^C]-pevonedistat 25 mg/m^2^

**Fig. 2:**
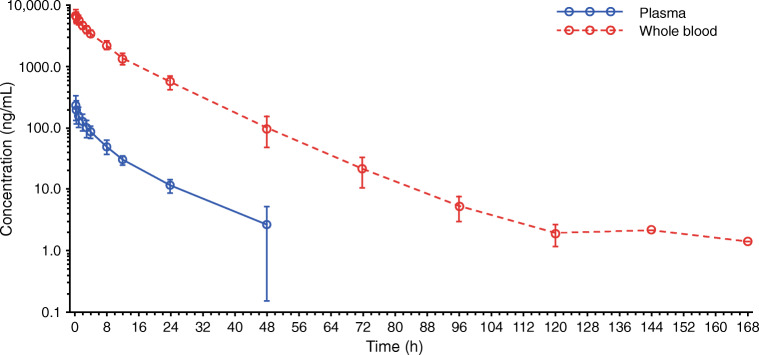
Mean concentration–time profiles of pevonedistat in plasma and whole blood following a single infusion of [^14^C]-pevonedistat 25 mg/m^2^

Following a single 1-h IV infusion of [^14^C]-pevonedistat 25 mg/m^2^, the mean t_1/2_ of pevonedistat and drug-related material (total radioactivity) in plasma was 8.4 and 15.6 h, respectively, and the mean t_1/2_ of pevonedistat and drug-related material in whole blood was 13.9 and 36.6 h, respectively. Figure 1 shows the mean concentration–time profiles of pevonedistat and drug-related material in plasma following the single dose of [^14^C]-pevonedistat. The longer t_1/2_ of drug-related material compared with pevonedistat that is observed in both plasma (Fig. 1) and whole blood (Supplementary Fig. S1) indicates elimination rate-limited clearance of the principal circulating pevonedistat metabolite(s). Figure 2 shows the mean concentration–time profile of pevonedistat in both plasma and whole blood following the single dose of [^14^C]-pevonedistat. There was a parallel log-linear decline of plasma and blood concentrations over the principal elimination phase, which was quantifiable in plasma up to 48 h post dose and in whole blood up to 120 h post dose.

Key PK parameters for pevonedistat and drug-related material in plasma are summarized in Table 2, and corresponding data in whole blood can be found in Supplementary Table S2. The mean exposure (AUC_∞_) ratio of pevonedistat versus total drug-related material in plasma and whole blood was 0.41 and 0.44, respectively, indicating that > 50% of the total radioactivity in circulation was accounted for by pevonedistat metabolites (Fig. 1 and Supplementary Fig. S1). The mean whole-blood-to-plasma ratio for pevonedistat AUC_∞_ was 40.8 (Table 2), suggesting a preferential distribution of pevonedistat in whole blood; this observation is supported by the observed concentration–time profiles of pevonedistat in plasma and whole blood (Fig. 2), which show substantially higher concentrations in whole blood versus plasma.

**Table 2:** Summary of PK parameters for pevonedistat and total radioactivity (drug-related material) in plasma following a single 1-h IV infusion of [^14^C]-pevonedistat 25 mg/m^2^

Parameter	Pevonedistat^a^	Drug-related material^a,b^
C_max_, ng/mL or ng eq/mL^c^	218 (43%)	277 (39%)
AUC_last_, h*ng/mL or h*ng eq/mL^c^	1409 (26%)	3421 (31%)
AUC_∞_, h*ng/mL or h*ng eq/mL^c^	1458 (27%)	3696 (30%)
t_1/2_, h^d^	8.4 (0.9)	15.6 (3.2)
CL, L/h^c^	32.2 (28%)	NA
V_ss_, L^c^	296 (36%)	NA
CL_R_, L/h^c^	0.8 (33%)	NA
Whole blood/plasma C_max_ ratio^d^	31.9 (8)	26.8 (6)
Whole blood/plasma AUC_last_ ratio^d^	42.2 (8)	38.6 (7)
Whole blood/plasma AUC_∞_ ratio^d^	40.8 (8)	36.8 (7)
Exposure ratio (AUC_∞_) of pevonedistat vs. total drug-related material	0.41	

*AUC*_***∞***_, AUC from time zero to infinity, calculated using the observed value of the last quantifiable concentration; *AUC*_*last*_, area under the concentration-time curve from time zero to time of the last quantifiable concentration; *CL*, clearance; *CL*_*R*_, renal clearance; *C*_*max*_, maximum observed concentration; *CV*, coefficient of variation; *IV*, intravenous; *NA*, not applicable; *PK*, pharmacokinetic; *SD*, standard deviation; *t*_*1/2*_, terminal half-life; *V*_*ss*_, volume of distribution at steady-state

^a^*N* = 7

^b^Drug-related material = Parent drug and all metabolites combined

^c^Geometric mean (CV)

^d^Mean (SD)

**Fig. 3:**
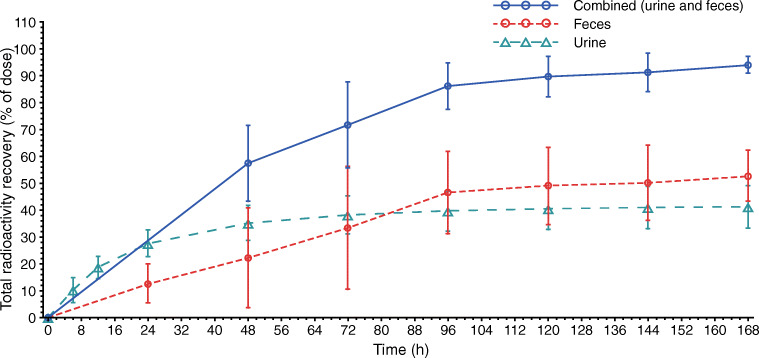
Mean time course of cumulative excretion of total radioactivity (drug-related material) in urine and feces following a single infusion of [^14^C]-pevonedistat 25 mg/m^2^

The geometric mean pevonedistat plasma CL was 32.2 L/h and geometric mean CL_R_ was 0.8 L/h (Table 2). Geometric mean whole blood CL was 0.81 L/h (Supplementary Table S2). Figure 3 shows the mean time course of the cumulative excretion of drug-related material in urine and feces. Following a single IV dose of [^14^C]-pevonedistat, 94.2% (± 3%) administered radioactivity was recovered in excreta (combined urine and feces), with 41% (± 7.9%) and 53% (± 9.5%) of administered radioactivity recovered in urine and feces, respectively, by 1 week post dose. The majority of the radioactivity was recovered by 96 h post dose.

### Safety

An overall summary of the safety profile following a single dose of [^14^C]-pevonedistat (part A) and after multiple doses of pevonedistat plus chemotherapy (part B) is presented in Table 3. Table 4 lists TEAEs reported in parts A and B.

**Table 3:** Summary of safety profile

Part A	Patients, *n* (%)		
	Single 1-hr IV infusion of [^14^C]-pevonedistat 25 mg/m^2^ (*N* = 8)		
TEAEs	4 (50.0)		
Drug-related	1 (12.5)		
Grade ≥ 3	1 (12.5)		
Grade ≥ 3 drug-related	0 (0.0)		
Serious TEAEs	1 (12.5)		
Drug-related	0 (0.0)		
Resulting in study discontinuation	0 (0.0)		
On-study deaths	1 (12.5)^a^		
Part B	Pevonedistat/carboplatin/paclitaxel (*n *= 5)	Pevonedistat/docetaxel (*n *= 2)	Total (*N *= 7)
TEAEs	5 (100.0)	2 (100.0)	7 (100.0)
Drug-related^b^	5 (100.0)	1 (50.0)	6 (85.7)
Grade ≥ 3	3 (60.0)	1 (50.0)	4 (57.1)
Grade ≥ 3 drug-related	2 (40.0)	0	2 (28.6)
Resulting in study drug discontinuation^b^	1 (20.0)	0	1 (14.3)
Serious TEAEs	2 (40.0)	1 (50.0)	3 (42.9)
Drug-related^b^	0	0	0
Resulting in study discontinuation^b^	1 (20.0)	0	1 (14.3)
On-study deaths	1 (20.0)	1 (50.0)	2 (28.6)

*TEAE*, treatment-emergent adverse event

^a^Patient consented for participation in part B but did not meet the entry criteria (laboratory values) and did not receive treatment. The patient died of sarcoma (disease progression) and is considered an on-study death for part A given that they died within 30 days of receiving the part A dose

^b^Related-ness of TEAEs refers to any drug in the study drug combination, as the causative agent (pevonedistat or standard of care) was not assigned for the study drug-related TEAEs reported. Additionally, discussion of TEAEs resulting in discontinuation of study drug refers to any drug in the study drug combination, as the causative agent (pevonedistat or standard of care) was not captured in the case report form

**Table 4:** All TEAEs (any grade and Grade ≥ 3) in part A and part B

Part A	Patients, *n* (%)					
	Single 1-hr IV infusion of [^14^C]-pevonedistat 25 mg/m^2^ (*N* = 8)					
	Any grade			Grade ≥ 3		
Sarcoma	1 (12.5)			1 (12.5)^a^		
Back pain	1 (12.5)			0		
Constipation	1 (12.5)			0		
Dizziness	1 (12.5)			0		
Urinary retention	1 (12.5)			0		
Vomiting	1 (12.5)			0		
Urinary retention	1 (12.5)			0		
Part B	Arm 1: Pevonedistat/ carboplatin/paclitaxel (*n* = 5)		Arm 2: Pevonedistat/ docetaxel (*n* = 2)		Total (*N* = 7)	
	All Grades	Grade ≥ 3	All Grades	Grade ≥ 3	All Grades	Grade ≥ 3
Vomiting	2 (40.0)	0	2 (100.0)	0	4 (57.1)	0
Nausea	2 (40.0)	0	1 (50.0)	0	3 (42.9)	0
Diarrhea	2 (40.0)	0	1 (50.0)	0	3 (42.9)	0
Asthenia	2 (40.0)	0	1 (50.0)	0	3 (42.9)	0
ALT increased	2 (40.0)	1 (20.0)	0	0	2 (28.6)	1 (14.3)
AST increased	2 (40.0)	1 (20.0)	0	0	2 (28.6)	1 (14.3)
Anemia	2 (40.0)	0	0	0	2 (28.6)	0
Fatigue	2 (40.0)	0	0	0	2 (28.6)	0
Thrombocytopenia	2 (40.0)	0	0	0	2 (28.6)	0
Pain	2 (40.0)	0	0	0	2 (28.6)	0
Pyrexia	1 (20.0)	0	1 (50.0)	0	2 (28.6)	0
Fibrosarcoma	0	0	1 (50.0)	1 (50.0)	1 (14.3)	1 (14.3)
General physical health deterioration	1 (20.0)	1 (20.0)	0	0	1 (14.3)	1 (14.3)
Platelet count decreased	1 (20.0)	1 (20.0)	0	0	1 (14.3)	1 (14.3)
Pneumonia	1 (20.0)	1 (20.0)	0	0	1 (14.3)	1 (14.3)
Abdominal distension	0	0	1 (50.0)	0	1 (14.3)	0
Abdominal pain	0	0	1 (50.0)	0	1 (14.3)	0
Abdominal pain upper	0	0	1 (50.0)	0	1 (14.3)	0
Alopecia	1 (20.0)	0	0	0	1 (14.3)	0
Arthralgia	0	0	1 (50.0)	0	1 (14.3)	0
Back pain	1 (20.0)	0	0	0	1 (14.3)	0
Blood bilirubin increased	1 (20.0)	0	0	0	1 (14.3)	0
Blood creatinine increased	1 (20.0)	0	0	0	1 (14.3)	0
Blood urea increased	1 (20.0)	0	0	0	1 (14.3)	0
Bone pain	0	0	1 (50.0)	0	1 (14.3)	0
Chest pain	1 (20.0)	0	0	0	1 (14.3)	0
Chills	1 (20.0)	0	0	0	1 (14.3)	0
Conjunctival hemorrhage	0	0	1 (50.0)	0	1 (14.3)	0
Dyspnea	1 (20.0)	0	0	0	1 (14.3)	0
Edema peripheral	0	0	1 (50.0)	0	1 (14.3)	0
Leukocytosis	1 (20.0)	0	0	0	1 (14.3)	0
Leukopenia	1 (20.0)	0	0	0	1 (14.3)	0
Mueller’s mixed tumor	1 (20.0)	0	0	0	1 (14.3)	0
Oral herpes	1 (20.0)	0	0	0	1 (14.3)	0
Peripheral venous disease	1 (20.0)	0	0	0	1 (14.3)	0
Rash	0	0	1 (50.0)	0	1 (14.3)	0
Spinal compression fracture	1 (20.0)	0	0	0	1 (14.3)	0
Swelling	1 (20.0)	0	0	0	1 (14.3)	0
Upper respiratory tract infection	1 (20.0)	0	0	0	1 (14.3)	0
Weight decreased	0	0	1 (50.0)	0	1 (14.3)	0

*ALT*, alanine aminotransferase; *AST*, aspartate aminotransferase; *TEAE*, treatment-emergent adverse event

^a^Serious TEAE of Grade 4 sarcoma resulted in the patient’s death one day after onset and was determined to be unrelated to treatment. All other TEAEs were Grade 1 or Grade 2 in intensity

^b^Patients with one or more TEAE within a level of Medical Dictionary for Regulatory Activities (MedDRA) Preferred Term were counted only once in that level

In part A, 4 patients (50%) experienced at least one TEAE (Table 4), among whom 1 patient (12.5%) had a TEAE that was determined by the investigator to be drug-related (Grade 1 dizziness). There were no serious TEAEs (SAEs). One patient with sarcoma died during the study after completing part A; the patient experienced an SAE of Grade 4 sarcoma (i.e., disease progression, determined to be unrelated to pevonedistat), which resulted in death 1 day after onset and 25 days after the part A pevonedistat dose.

All 7 patients in part B experienced at least one TEAE; the most common were vomiting, which occurred in 4 patients (57.1%) overall, and nausea, diarrhea and asthenia in 3 patients (42.9%) overall (Table 4). At least one drug-related TEAE was experienced by 6 patients (85.7%) overall (all 5 patients in the pevonedistat/carboplatin/paclitaxel arm and 1 of 2 patients in the pevonedistat/docetaxel arm). The most common drug-related TEAE was vomiting, which occurred in 3 patients (42.9%) overall (2 patients in the pevonedistat/carboplatin/paclitaxel arm and 1 patient in the pevonedistat/docetaxel arm), and 2 patients overall experienced increased alanine aminotransferase (ALT), increased aspartate aminotransferase (AST), nausea (all in the pevonedistat/carboplatin/paclitaxel arm), and asthenia (1 patient in each arm). Four patients (57.1%) in part B had Grade ≥ 3 TEAEs, which were considered to be drug-related in 2 patients (28.6%; increased ALT and AST in 1 patient, and decreased platelet count in 1 patient). One patient in the pevonedistat/carboplatin/paclitaxel arm had a TEAE of Grade 3 pneumonia resulting in study drug discontinuation. Three patients (42.9%) had at least one SAE, none of which were considered related to treatment; 2 were receiving pevonedistat/carboplatin/paclitaxel and 1 was being treated with pevonedistat/docetaxel. Two on-study deaths occurred in part B, neither of which were attributed to any of the study drugs. The deaths were due to general physical health deterioration in a patient receiving pevonedistat/carboplatin/paclitaxel, and disease progression 9 days after the last dose in a patient receiving pevonedistat/docetaxel.

### Efficacy

In part B, all 7 patients were evaluable for response. One patient achieved a best response of partial response (per RECIST version 1.1), and 5 patients achieved a best response of stable disease. The remaining patient experienced progressive disease. All 7 patients subsequently discontinued treatment due to progressive disease (*n* = 6) or an SAE (*n* = 1; pneumonia).

## Discussion

Evaluation of the effects of intrinsic (e.g., functional capacity of eliminating organs) and extrinsic (e.g., drug–drug interactions) factors on PK is a crucial component of the clinical pharmacology characterization of anticancer agents to inform appropriate dosing and administration across patient populations and clinical contexts of use [18, 19]. Radiolabeled mass balance studies play an important role in quantitatively defining routes of elimination and clearance mechanisms, thereby providing valuable inputs to inform a scientifically guided and rational approach to evaluating intrinsic/extrinsic factor effects on PK [20].This study evaluated the mass balance, PK, routes of elimination, and safety of the NAE inhibitor pevonedistat in 8 patients with advanced solid tumors. In summary, results show that following a single IV infusion of [^14^C]-pevonedistat 25 mg/m^2^, the mean t_1/2_ in plasma was 8.4 h for pevonedistat and 15.6 h for drug-related material. The half-life of pevonedistat in plasma was similar to that previously reported in patients with advanced malignancies [11]. In patients with metastatic melanoma, pevonedistat previously demonstrated an elimination half-life of approximately 10 hours in plasma [11]. In this analysis, corresponding mean t_1/2_ values in whole blood were 13.9 h for pevonedistat and 36.6 h for drug-related material. Visual inspection of semi-logarithmic plots of overlaid plasma and whole blood concentration–time profiles of pevonedistat indicated a parallel log-linear decline in drug concentrations in the two compartments, supporting the inference of comparable disposition kinetics in blood and plasma.

The observed mean whole-blood-to-plasma ratio of pevonedistat AUC_∞_ was 40.8, suggesting a preferential distribution of pevonedistat in whole blood. The preferential distribution to red blood cells is considered to be due to binding to carbonic anhydrase II (CAII) (Takeda data on file), an intracellular enzyme abundant in human erythrocytes [21, 22]. This may explain the extensive partitioning of pevonedistat in whole blood that has previously been observed in animal species (Takeda data on file) and humans. Exposure (AUC_∞_) ratios of pevonedistat versus total drug-related material in our analysis were 0.41 and 0.44 in plasma and whole blood, respectively, indicating that > 50% of the total radioactivity in circulation was accounted for by pevonedistat metabolites.

The mean plasma CL of pevonedistat was 32.2 L/h in this study. This value is consistent with that reported in a previous population PK analysis in more than 300 patients, in which the typical CL of pevonedistat was estimated to be 31.5 L/h [17]. Mean whole blood CL of pevonedistat was 0.81 L/h in our analysis and was approximately 1% of hepatic blood flow. These data support the conclusion that pevonedistat is a low clearance drug. In terms of excretion kinetics, at 1 week post dose, an average of 41% and 53% of administered radioactivity was recovered in the urine and feces of patients, respectively. This translates to a cumulative 94% recovery of the administered radioactive dose 1 week after drug administration.

The CL_R_ of pevonedistat was 0.8 L/h. This represents approximately 2.5% of pevonedistat total plasma clearance, indicating that the renal elimination pathway played a minor role in the clearance of pevonedistat. As a corollary, given that these data are following IV administration, it may be inferred that metabolic clearance represents the primary route of disposition of pevonedistat in humans. This is consistent with the finding from the population PK analysis that mild or moderate renal impairment (creatinine clearance 30–89 mL/min) does not have a clinically meaningful impact on pevonedistat exposures [17]. Therefore, based on PK considerations, no pevonedistat dose modifications are expected to be required in patients with mild-to-moderate renal impairment. It should be noted that the effect of severe renal impairment on pevonedistat is not yet known and warrants further investigation [17]. The presence of circulating metabolites, the minor contribution of CL_R_ to pevonedistat disposition, and the predominantly urinary (non-parent drug) and fecal elimination of drug-related material suggest that hepatic metabolism plays a major role in the overall clearance of pevonedistat. Accordingly, a phase I/Ib study (NCT03814005) is currently ongoing to evaluate the effect of mild hepatic impairment on pevonedistat PK in patients with higher-risk myelodysplastic syndromes, chronic myelomonocytic leukemia, or AML. The effect of severe renal impairment will also be examined. Results from these assessments will inform pevonedistat dosing recommendations in these special patient populations. Although pevonedistat was identified to be a substrate of CYP3A in vitro, the results of a clinical drug–drug interaction study did not indicate clinically meaningful alterations in its PK upon co-administration with the strong CYP3A inhibitor itraconazole [23]. The results of the current mass balance study, future metabolite profiling results on excreta collected in this study, and complementary follow-up in vitro metabolism/transport studies will be crucial in providing a holistic understanding of the principal mechanistic and molecular determinants of pevonedistat disposition.

The safety profile of pevonedistat in this study was consistent with prior clinical experience. No new safety signals were observed in our analysis. In part A, dizziness was the only reported TEAE that was considered to be drug-related. In part B, the most common drug-related TEAE reported was vomiting, which occurred in 3 patients. Grade ≥ 3 increased ALT, increased AST, and decreased platelet count occurring in part B were considered to be related to treatment. Overall, the safety profile during part B of this study was consistent with what was observed in a previous study in which pevonedistat was administered with standard-of-care chemotherapy [12].

In conclusion, the results of this radiolabeled mass balance study of the NAE inhibitor pevonedistat following a single 1-h infusion of [^14^C]-pevonedistat 25 mg/m^2^ in cancer patients confirm a predominant role for metabolic clearance and a minor role of renal clearance in the disposition of this agent. Recovery of radioactivity was nearly complete (94%) in 1 week following single-dose IV administration. PK data support low systemic clearance, presence of circulating metabolite(s), and extensive whole blood partitioning of pevonedistat and total drug-related material. Further analyses of metabolite profiles in plasma and excreta collected in this study will provide greater molecular resolution of the biotransformation pathways of pevonedistat in humans. These findings, taken together, provide important information to enable rational assessment of the impact of intrinsic and extrinsic factors on pevonedistat exposure as part of the ongoing clinical pharmacology evaluations in the development of this investigational anticancer agent.

## Electronic supplementary material

ESM 1 (PDF 574 kb)

## Data Availability

The datasets, including the redacted study protocol, redacted statistical analysis plan, and individual participants data supporting the results reported in this article, will be made available within three months from initial request, to researchers who provide a methodologically sound proposal. The data will be provided after its de-identification, in compliance with applicable privacy laws, data protection and requirements for consent and anonymization.
