# Supplementary material for: Phase I study assessing the mass balance, pharmacokinetics, and excretion of [14C]-pevonedistat, a NEDD8-activating enzyme inhibitor in patients with advanced solid tumors
Source: Invest New Drugs. 2020 Oct 22;39(2):488–98. doi: 10.1007/s10637-020-01017-x (PMC7960626; doi:10.1007/s10637-020-01017-x)

Electronic supplementary material – online only

Supplementary methods

Inclusion criteria

Each patient was required to meet the following inclusion criteria to be enrolled in the study:

1. Male or female patients aged 18 years or older.
2. Patients must have had a histologically or cytologically confirmed metastatic or locally advanced and incurable solid tumor that was felt to be appropriate for treatment with one of the two chemotherapy regimens in part B of this study (carboplatin + paclitaxel or docetaxel), or had progressed despite prior standard therapy, or for whom conventional therapy was not considered effective. The tumor must have been radiographically or clinically evaluable and/or measurable.
3. Eastern Cooperative Oncology Group (ECOG) performance status of 0 to 1.
4. Expected survival longer than 3 months from enrollment in the study.
5. Recovered (i.e., Grade ≤1 toxicity) from the effects of prior antineoplastic therapy.
6. Clinical laboratory values as specified below within 3 days before the first dose of study drug:
   - Hemoglobin ≥9 g/dL. Patients may have been transfused to achieve this value.
   - Total bilirubin ≤upper limit of the normal range (ULN).
   - Alanine aminotransferase (ALT), aspartate aminotransferase (AST), and alkaline phosphatase (ALP) ≤2.5×ULN.
   - For patients to be treated with pevonedistat/docetaxel in part B, AST and ALT were required to be ≤1.5×ULN and total bilirubin was required to be within the normal range.
   - Calculated creatinine clearance ≥50 mL/min.
   - Absolute neutrophil count (ANC) ≥1,500/mm^3.
   - Platelet count ≥100,000/mm^3.
   - Prothrombin time and activated partial thromboplastin time ≤1.5×ULN.
   - Albumin ≥2.7 g/dL.
7. Suitable venous access for the study-required blood sampling (including pharmacokinetic [PK] sampling).
8. Female patients who:
   - Were postmenopausal for at least 1 year before the Screening visit, or
   - Were surgically sterile, or

- If they were of childbearing potential, they and their male partners agreed to practice 1 highly effective method and 1 additional effective (barrier) method of contraception at the same time, from the time of signing the informed consent through 4 months after the last dose of study drug, or
- Agreed to practice true abstinence, when this was in line with the preferred and usual lifestyle of the patient. (Periodic abstinence [e.g. calendar, ovulation, symptothermal, postovulation methods] withdrawal, spermicides only, and lactational amenorrhea were not acceptable methods of contraception. Female and male condoms were not to be used together).

9. Male patients, even if surgically sterilized (i.e., status post-vasectomy), who:
   - Agreed to practice effective barrier contraception during the entire study treatment period
   - and through 4 months after the last dose of study drug, or
   - Agreed to practice true abstinence, when this was in line with the preferred and usual lifestyle of the patient. (Periodic abstinence [e.g. calendar, ovulation, symptothermal, postovulation methods for the female partner] withdrawal, spermicides only, and lactational amenorrhea were not acceptable methods of contraception. Female and male condoms were not to be used together).
10. Patients who were willing to refrain from donating blood for at least 90 days after their dose of pevonedistat and (for male patients) willing to refrain from donating semen for at least 4 months after their dose of pevonedistat.
11. Voluntary written consent must have been given before performance of any study related procedure not part of standard medical care, with the understanding that consent could be withdrawn by the patient at any time without prejudice to future medical care.

Exclusion criteria

Patients who met any of the following exclusion criteria were not to be enrolled in the study:

1. Inability to comply with study visits and procedures including required inpatient confinement.
2. Patient had irregular defecation patterns (<1 defecation per 2 days or excessive diarrhea) and/or had a history of changes in bowel habits with daily routine or environment changes.
3. Treatment with any systemic antineoplastic therapy or any investigational products within 21 days before the first dose of study treatment.
4. Major surgery within 14 days before the first dose of study treatment or scheduled surgery during part A of the study.

5. Receiving antibiotic therapy within 14 days before the first dose of study treatment.
6. Radiotherapy within 14 days before the first dose of study treatment.
7. Prior treatment with radiation therapy involving ≥25% of the hematopoietically active bone marrow.
8. Treatment with moderate or strong CYP3A inhibitors or inducers within 14 days before the first dose of pevonedistat. Patients must have had no history of amiodarone use in the 6 months before the first dose of pevonedistat.
9. Prior treatment with pevonedistat; however, prior treatment with docetaxel, paclitaxel, and carboplatin was allowed.
10. Known hypersensitivity or history of severe intolerance or toxicity to chemotherapeutic agents including known history of severe hypersensitivity reactions to docetaxel (polysorbate 80-based formulations) for patients to be treated with pevonedistat/docetaxel; history of hypersensitivity to carboplatin for patients to be treated with pevonedistat/carboplatin/paclitaxel; or history of severe hypersensitivity to paclitaxel (Cremophor-based formulations) for patients to be treated with pevonedistat/carboplatin/paclitaxel.
11. Life-threatening illness or serious (acute or chronic) medical or psychiatric illness unrelated to cancer that could have increased the risk associated with trial participation or investigational product administration or could have interfered with the interpretation of trial results or, in the investigator’s opinion, could have potentially interfered with the completion of treatment according to the protocol.
12. Active, uncontrolled infection or severe infectious disease, such as severe pneumonia, meningitis, septicemia, or methicillin-resistant *Staphylococcus aureus* infection within 2 weeks before dosing.
13. Known human immunodeficiency virus (HIV) seropositive or known hepatitis B surface antigen seropositive or known or suspected active hepatitis C infection. Note: patients who had isolated positive hepatitis B core antibody (i.e., in the setting of negative hepatitis B surface antigen and negative hepatitis B surface antibody) must have had an undetectable hepatitis B viral load.
14. History of urinary and/or fecal incontinence.
15. Persistent diarrhea (Grade ≥2) lasting >3 days within 2 weeks before the first dose of study treatment.
16. Clinically significant central nervous system disease defined as newly diagnosed, untreated, progressive, or requiring steroids for control of symptoms.
17. Newly diagnosed or uncontrolled cancer-related central nervous system disease.
18. Known hepatic cirrhosis or severe pre-existing hepatic impairment.

19. Uncontrolled high blood pressure (i.e., systolic blood pressure >180 mm Hg, diastolic blood pressure >95 mm Hg).
20. Left ventricular ejection fraction <50% as assessed by echocardiogram or radionuclide angiography.
21. Patients with ischemic heart disease who had acute coronary syndrome, myocardial infarction, or revascularization (e.g., coronary artery bypass graft, stent) in the past 6 months were excluded. However, patients with ischemic heart disease who had acute coronary syndrome, myocardial infarction, or revascularization greater than 6 months before screening and who were without cardiac symptoms could enroll. In addition, patients with congestive heart failure (New York Heart Association Class III or IV) or New York Heart Association Class II with recent decompensation requiring hospitalization within 4 weeks before screening and patients with severe pulmonary arterial hypertension could enroll.
22. Arrhythmia (e.g., history of polymorphic ventricular fibrillation or torsade de pointes, permanent atrial fibrillation defined as continuous atrial fibrillation for ≥6 months, and persistent atrial fibrillation, defined as sustained atrial fibrillation lasting 7 days and/or requiring cardioversion in the last 4 weeks before Screening). However, patients with Grade <3 atrial fibrillation for a period of at least 6 months could enroll. Grade 3 atrial fibrillation was defined as symptomatic and incompletely controlled medically, or controlled with device (e.g., pacemaker) or ablation, and was excluded. Patients with paroxysmal atrial fibrillation were permitted to enroll.
23. Prolonged rate-corrected QT interval (QTc) ≥500 msec, calculated according to institutional guidelines.
24. Implantable cardioverter defibrillator.
25. Patients with a cardiac pacemaker whose heart rate was set at a fixed rate and patients on concomitant medication that could have limited increase in heart rate in response to hypotension (e.g., high-dose beta blocker).
26. Moderate to severe aortic stenosis, moderate to severe mitral stenosis, or other valvulopathy (ongoing).
27. Known moderate to severe chronic obstructive pulmonary disease, interstitial lung disease, pulmonary fibrosis, or pulmonary arterial hypotension.
28. Female patients who were lactating and breastfeeding or who had a positive serum pregnancy test during the screening period or a positive urine pregnancy test on day 1 before the first dose of study drug.
29. Female patients who intended to donate eggs (ova) during the course of this study or 4 months after receiving their last dose of study drug.

30. Male patients who intended to donate sperm during the course of this study or 4 months after receiving their last dose of study drug.
31. Patients who required chronic treatment with breast cancer resistance protein (BCRP) or P-glycoprotein (P-gp) inhibitors.

Entry criteria for continuation into part B

To be eligible for part B, patients were required to meet the following entry criteria:

- ECOG performance status of 0 to 1.
- Laboratory values for hemoglobin, ANC, platelets, total bilirubin, ALT, AST, ALP, and serum creatinine or calculated/measured creatinine clearance as specified in the main inclusion criteria.
- Diarrhea symptoms resolved to Grade 1 or better.
- QTc interval <500 msec.
- Computed tomography (CT) scan or magnetic resonance imaging (MRI) of the chest, abdomen, and pelvis within 28 days of cycle 1 day 1.

Bioanalytical assay methods

All samples were analyzed applying the methods described below:

Method for plasma

- Validated range: 30.0–80,000 dpm/mL
- Sample preparation:
    - An aliquot of 250 µL of the plasma sample was transferred into a 7 mL glass vial (Perkin Elmer, NL), and 2.5 mL scintillation cocktail (Ultima Gold™ [Perkin Elmer, NL]) was added. After vortex mixing for at least 5 sec, the sample was placed in the liquid scintillation counter for at least 30 min prior to counting.

Method for whole blood

- Validated range: 50.0–20,000 dpm/mL
- Sample preparation:

- An aliquot of 300 µL of the whole blood sample was transferred into a 20 mL glass vial. An amount of 1.00 mL of tissue solubilizer (Solvable™, Perkin Elmer, NL) was added and the sample was incubated for 60 min at 60°C in a water bath. After cooling in a cold water bath, 100 µL of 0.1 M titriplex (VWR, NL) was added and the sample was decolorized by adding 3 times a volume of 75 µL hydrogen peroxide (VWR, NL) in steps of 5 min. After incubation for 15 min at room temperature, the mixture was heated again for 15 min at 45°C in a water bath, followed by 30 min at 60°C. After cooling in a cold water bath, 18 mL of scintillation cocktail (Ultima Gold™ [Perkin Elmer, NL]) was added. After vortex mixing for at least 5 sec, the vial was placed in an ultra-sonication bath at room temperature for 5 min. The vial was placed in the liquid scintillation counter for at least 35 h prior to counting.

Method for urine

- Validated range: 10.0–1,000,000 dpm/mL
- Sample preparation:
    - An aliquot of 1000 µL of the urine sample was transferred into a 7 mL glass vial (Perkin Elmer, NL) and 5 mL scintillation cocktail (Ultima Gold™ [Perkin Elmer, NL]) was added. After vortex mixing for at least 5 sec, the sample was placed in the liquid scintillation counter for at least 30 min before counting.

Method for feces homogenization in water

- Preparation of homogenate:
    - The feces samples were quantitatively transferred into a container per subject and per 24 h interval. A minimum of water (1 to 2 weight equivalents) was added and recorded. The samples were homogenized with an Ultra Turrax® mixer by mixing for at least 2 min.

- After homogenization:
  - 2 x 15 g aliquots (Feces Total Radioactivity Set 1 and Set 2) were taken for determination of total ^{14}C-radioactivity and quick count analysis and were stored at a nominal -70°C (except for quick counts).
  - 2 x 40 g aliquots for metabolic profiling (Feces MetPro Set 1 and Set 2) were stored at a nominal -70°C.
  - 1 x 150 g aliquot feces for reserve (For reserve) for metabolite profiling were stored at a nominal -70°C.
- The remainders of the homogenates were discarded.

Method for feces

- Validated range: 40.0–1,000,000 dpm/g
- Sample preparation:
  - After homogenization of the feces samples, an accurately weighed aliquot of approximately 500 mg of the feces homogenate sample was dried in a stove at +50°C for at least 3 h. After the addition of 100 µL Combustaid™ (Perkin Elmer, NL) to the dry homogenates, the sample was combusted in a Sample Oxidizer Model 307 (Perkin Elmer, NL). 7 mL CarboSorb-E™ (Perkin Elmer, NL) was used as an absorber agent for carbon dioxide. At the end of the combustion cycle the absorber was mixed with 13 mL of the scintillant PermaFluor E™ (Perkin Elmer, NL). The sample was placed in the liquid scintillation counter for at least 30 min prior to counting.

Method for vomit

- Note: Based on the sample composition (clear or not clear solution) the urine or feces method could be selected for analysis. For the samples collected in this study, the urine method was used.

- Validated range: 10.0–1,000,000 dpm/mL
- Sample preparation with urine method:
    - An aliquot of 1000 µL (weight was recorded) of the vomit sample was transferred into a 7 mL glass vial (Perkin Elmer, NL) and 5 mL scintillation cocktail (Ultima Gold™ [Perkin Elmer, NL]) was added. After vortex mixing for at least 5 sec, the sample was placed in the liquid scintillation counter for at least 30 min before counting.

Supplementary Tables/Figures

Table S1 Pharmacokinetic parameter estimates

**Parameters**	**Definition**	**Units**
Total radioactivity in plasma and whole blood:		
C_{max}	Maximum observed total radioactivity	ng eq/mL
t_{max}	First time at which C_{max} occurs	h
AUC_{last}	Area under the total radioactivity–time curve from time 0 to the time of the last quantifiable measurement, estimated using the linear-log trapezoidal rule	h*ng eq/mL
$t_{1/2z}$	Terminal disposition phase half-life	h
AUC_{∞}	Area under the total radioactivity–time curve from time 0 to infinity	h*ng eq/mL
Total radioactivity in urine:		
Ae_{urine}, $^{14}C_{,t1\text{-}t2}$	Amount of [^{14}C]-radioactivity excreted into urine per sampling interval	ng eq
Fe_{urine}, $^{14}C_{,t1\text{-}t2}$	Fraction of administered [^{14}C]-radioactivity excreted in urine per sampling interval	%
Ae_{urine}, ^{14}C	Cumulative amount of [^{14}C]-radioactivity excreted into urine up to the last sampling interval	ng eq
Fe_{urine}, ^{14}C	Cumulative fraction of administered [^{14}C]-radioactivity excreted in urine	%
Total radioactivity in feces:		
Ae_{feces}, $^{14}C_{,t1\text{-}t2}$	Amount of [^{14}C]-radioactivity excreted into feces per sampling interval	ng eq
Fe_{feces}, $^{14}C_{,t1\text{-}t2}$	Fraction of administered [^{14}C]-radioactivity excreted in feces per sampling interval	%

Ae_{feces}, ^{14}C	Cumulative amount of [^{14}C]-radioactivity excreted into feces up to the last sampling interval	ng eq
Fe_{feces}, ^{14}C	Cumulative fraction of the administered [^{14}C]-radioactivity excreted in feces	%
Total cumulative excretion of total radioactivity:		
$Ae_{total,}$ ^{14}C	Total cumulative excretion of [^{14}C]-radioactivity excreted in urine and feces: $Ae_{total,}$ ^{14}C = $Ae_{urine,}$ ^{14}C + $Ae_{feces,}$ ^{14}C	ng eq
Fe_{total}, ^{14}C	Cumulative fraction of administered [^{14}C]-radioactivity excreted in urine and feces: $Fe_{total,}$ ^{14}C = Fe_{urine}, ^{14}C + Fe_{feces}, ^{14}C	%
Pevonedistat in whole blood and plasma:		
C_{max}	Maximum observed pevonedistat concentration	ng/mL
T_{max}	First time at which C_{max} occurs	h
AUC_{last}	Area under the concentration–time curve from time 0 to the time of the last quantifiable measurement, estimated using the linear-log trapezoidal rule	h*ng/mL
$t_{1/2z}$	Terminal disposition phase half-life	h
AUC_{∞}	Area under the concentration–time curve from time 0 to infinity	h*ng/mL
CL	Clearance	L/h
Pevonedistat in urine (per sampling interval and total):		
Ae_{urine}	Amount of pevonedistat excreted unchanged in urine	µg
Cumulative Ae_{urine}	Cumulative amount of pevonedistat excreted unchanged in urine	µg

Fe_{urine}	Fraction of the administered dose excreted unchanged in urine	%
Cumulative Fe_{urine}	Cumulative fraction of the administered dose excreted unchanged in urine	%
CL_R	Renal clearance	L/h

Table S2 Summary of PK parameters for pevonedistat and total radioactivity (drug-related material) in whole blood following a single 1-hr IV infusion of [^{14}C]-pevonedistat 25 mg/m^2

Parameter	Pevonedistat[a]	Drug-related material[a,b]
$C_{max,}$ ng/mL or ng eq/mL[c]	6724 (23%)	7250 (17%)
$AUC_{last,}$ h*ng/mL or h*ng eq/mL[c]	58545 (17%)	129683 (25%)
$AUC_{\infty,}$ h*ng/mL or h*ng eq/mL[c]	58577 (17%)	133711 (26%)
$t_{1/2,}$ h[d]	13.9 (5.4)	36.6 (4.5)
CL, L/h[c]	0.81 (17%)	NA
$V_{ss,}$ L[c]	9.1 (16%)	NA

AUC_{∞}, AUC from time zero to infinity, calculated using the observed value of the last quantifiable concentration; *AUC_{last}*, area under the concentration–time curve from time zero to time of the last quantifiable concentration; *CL*, clearance; *C_{max}*, maximum observed concentration; *CV*, coefficient of variation; *NA*, not applicable; *PK*, pharmacokinetic; $t_{1/2}$, terminal half-life; *V_{ss}*, volume of distribution at steady-state.

[a]*N* = 7.

[b]Drug-related material = Parent drug and all metabolites combined.

[c]Geometric mean (CV)

[d]Mean (SD)

Fig. S1 Mean concentration–time profiles of pevonedistat and total radioactivity in whole blood following a single infusion of [^{14}C]-pevonedistat 25 mg/m^2

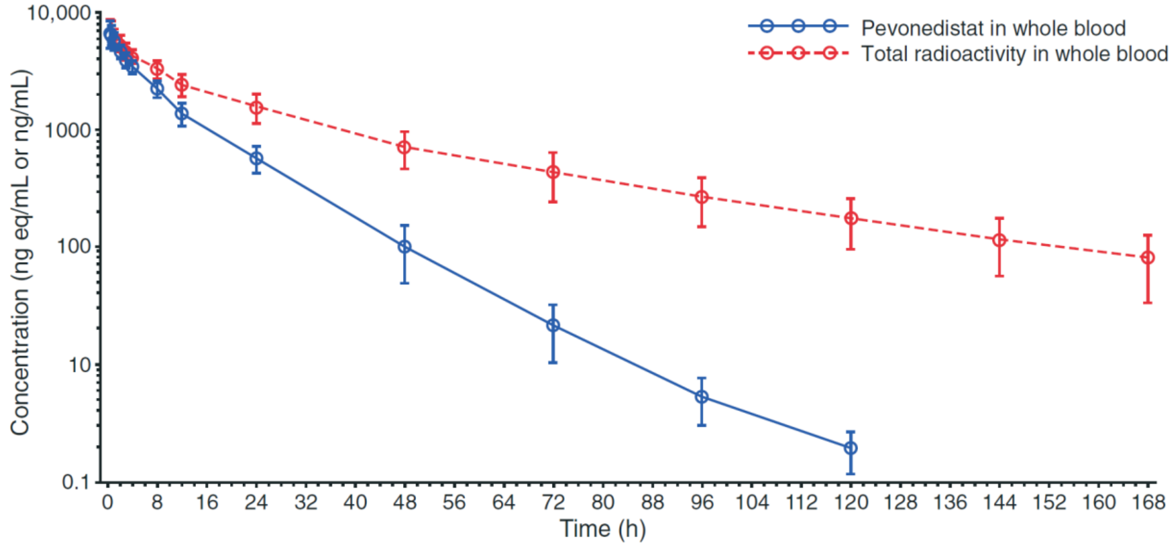

Supplement: Supplementary file 1 — (PDF 574 kb) [file 10637_2020_1017_MOESM1_ESM.pdf]
